# Comparative genome analysis of 24 bovine-associated * Staphylococcus* isolates with special focus on the putative virulence genes

**DOI:** 10.7717/peerj.4560

**Published:** 2018-03-30

**Authors:** Silja Åvall-Jääskeläinen, Suvi Taponen, Ravi Kant, Lars Paulin, Jochen Blom, Airi Palva, Joanna Koort

**Affiliations:** 1 Department of Veterinary Biosciences, Division of Microbiology and Epidemiology, Faculty of Veterinary Medicine, University of Helsinki, Helsinki, Finland; 2 Department of Production Animal Medicine, Faculty of Veterinary Medicine, University of Helsinki, Helsinki, Finland; 3 Institute of Biotechnology, University of Helsinki, Helsinki, Finland; 4 Bioinformatics and Systems Biology, Justus Liebig Universität Gießen, Gießen, Germany

**Keywords:** *Staphylococcus*, Comparison, Virulence factor, Non-aureus staphylococci

## Abstract

Non-aureus staphylococci (NAS) are most commonly isolated from subclinical mastitis. Different NAS species may, however, have diverse effects on the inflammatory response in the udder. We determined the genome sequences of 20 staphylococcal isolates from clinical or subclinical bovine mastitis, belonging to the NAS species *Staphylococcus agnetis, S. chromogenes,* and *S. simulans*, and focused on the putative virulence factor genes present in the genomes. For comparison we used our previously published genome sequences of four *S. aureus* isolates from bovine mastitis. The pan-genome and core genomes of the non-aureus isolates were characterized. After that, putative virulence factor orthologues were searched *in silico*. We compared the presence of putative virulence factors in the NAS species and *S. aureus* and evaluated the potential association between bacterial genotype and type of mastitis (clinical vs. subclinical). The NAS isolates had much less virulence gene orthologues than the *S. aureus* isolates. One third of the virulence genes were detected only in *S. aureus*. About 100 virulence genes were present in all *S. aureus* isolates, compared to about 40 to 50 in each NAS isolate. *S. simulans* differed the most. Several of the virulence genes detected among NAS were harbored only by *S. simulans*, but it also lacked a number of genes present both in *S. agnetis* and *S. chromogenes*. The type of mastitis was not associated with any specific virulence gene profile. It seems that the virulence gene profiles or cumulative number of different virulence genes are not directly associated with the type of mastitis (clinical or subclinical), indicating that host derived factors such as the immune status play a pivotal role in the manifestation of mastitis.

## Introduction

In dairy production, *Staphylococcus aureus* is a major pathogen causing mastitis in dairy cows (Bramley & Dodd, 1984). *S. aureus* is well-known for its ability to cause severe clinical mastitis, but most often causes chronic subclinical mastitis. It causes a moderate to strong increase in milk somatic cell count (SCC) thereby impairing milk quality (Bramley & Dodd, 1984). Non-aureus staphylococci (NAS), including both coagulase-negative and coagulase-variable species, are considered minor pathogens, which usually cause subclinical or mild clinical bovine mastitis with only a moderate increase in milk SCC (Taponen & Pyörälä, 2009). The importance of NAS as cause of mastitis is debated, as some researchers consider them true mastitis pathogens (Taponen et al., 2006; Taponen et al., 2007; Supré et al., 2011), while others consider them harmless contaminants or even beneficial since NAS may protect the udder against major pathogens (De Vliegher et al., 2003). Non-aureus staphylococci, however, are the bacteria most frequently isolated in mastitic milk samples in many countries (De Vliegher et al., 2012; Zuniga et al., 2015; Taponen et al., 2017). Prevalence of NAS intramammary infection (IMI) is especially high around first calving (Taponen et al., 2006; Sampimon et al., 2009a; Tenhagen et al., 2009). Part of the NAS IMIs around calving cure spontaneously but others persist for long periods during the entire lactation or even over the dry period (Taponen et al., 2007; Rajala-Schultz et al., 2009).

About 20 NAS species have been isolated in bovine milk samples (Taponen et al., 2007; Sampimon et al., 2009b; Park et al., 2011; Persson Waller et al., 2011; Piessens et al., 2011; Supré et al., 2011; Mørk et al., 2012; Quirk et al., 2012), but five species predominate, namely *Staphylococcus chromogenes, S. epidermidis, S. haemolyticus, S. simulans*, and *S. xylosus*. Others are only occasionally isolated (Vanderhaeghen et al., 2014). Although NAS causing bovine mastitis are treated as a group, differences exist between them. Some evidence shows that different NAS species or strains can have diverse effects on the inflammatory response in the udder (Simojoki et al., 2011; Supré et al., 2011; Åvall-Jääskeläinen et al., 2013; Fry et al., 2014b; Savijoki et al., 2014; Piccart et al., 2016).

Non-aureus staphylococci are natural microbiota of bovine skin. This seems to be true for at least *S. chromogenes*, which is most frequently isolated in bovine IMI in almost all studies and geographical locations (Sampimon et al., 2009b; Mørk et al., 2012; Fry et al., 2014b). Several studies isolated it from teat skin and apex and other parts of heifers and cows (De Vliegher et al., 2003; Taponen, Björkroth & Pyörälä, 2008). It seems to be present in all herds (Piessens et al., 2011; Supré et al., 2011; Mørk et al., 2012; Bexiga et al., 2014) and is much more common in primiparous than multiparous cows (Taponen et al., 2006; Thorberg et al., 2009; Mørk et al., 2012).

On the contrary, the two second most common NAS species, *S. epidermidis* and *S. simulans,* are more common in IMIs of multiparous cows (Taponen et al., 2006; Thorberg et al., 2009; Mørk et al., 2012). They are suspected to transmit from cow to cow, possibly during milking via milking equipment. These NAS species are frequently isolated and cause IMI in certain herds, but are only occasionally found or not detected in others (Gillespie et al., 2009; Thorberg et al., 2009; Piessens et al., 2011; Supré et al., 2011; Bexiga et al., 2014). *S. simulans* tends to cause a stronger inflammatory reaction than many other NAS species (Taponen et al., 2006; Simojoki et al., 2011).

*S. agnetis*, a bovine-associated coagulase-variable species (Taponen et al., 2012), is closely related to and was earlier classified as *S. hyicus*. *S. agnetis* is frequently isolated from mastitic milk samples, but does not belong to the most commonly isolated NAS species (Taponen et al., 2016; Adkins et al., 2017). *S. hyicus* reportedly causes stronger inflammatory reactions in the udder than many other NAS species (Myllys, 1995). Because approximately more than half of the isolates classified as *S. hyicus,* based on phenotypic speciation methods, were reclassified as *S. agnetis* using more precise molecular methods (Adkins et al., 2017), it is likely that at least part of the *S. agnetis* isolates also have the capacity to cause a significant inflammatory reaction in the udder. Recently, *S. agnetis* was also shown to be the causative agent in bacterial chondronecrosis with osteomyelitis leading to lameness in broiler chickens in the wire floor system (Al-Rubaye et al., 2015) and a potential causative agent in endocarditis and septicemia in broiler breeders (Poulsen et al., 2017).

We aimed to compare three different NAS species and *S. aureus* in terms of the presence of genes encoding putative virulence factors and to evaluate the potential association between bacterial genotype and type of bovine mastitis (clinical vs. subclinical). Twenty staphylococcal isolates, belonging to three NAS species, *S. agnetis, S. chromogenes,* and *S. simulans,* were genome sequenced. For comparative genomics, we also included previously published sequences of four *S. aureus* isolates (Kant et al., 2015). The pangenome (the entire gene set of all isolates) and core genomes (number of genes present in each isolate) of these non-aureus isolates were characterized. All the isolates, including the *S. aureus* isolates, originate from a mastitis treatment study (Taponen et al., 2006). In each species, half of the isolates selected for this study were from mastitis cases with mild to moderate clinical signs including changes in milk appearance, local and/or systemic signs (clinical mastitis) and half from cases without clinical signs (subclinical mastitis). The presence of putative virulence genes was studied and compared between the staphylococcal species as well as between the mastitis types. We chose these NAS species because: (1) *S. chromogenes* is the most common NAS species isolated in bovine milk and is especially common in udders of heifers around calving (Rajala-Schulz et al., 2004), (2) *S. simulans* is commonly isolated in mastitic milk samples especially in Nordic countries (Waage et al., 1999; Taponen et al., 2006; Capurro et al., 2009), and (3) *S. agnetis,* described by our group, still needs a more detailed description of its characteristics (Taponen et al., 2012).

## Materials & Methods

### Bacterial isolates, growth conditions and DNA extraction

Twenty-four staphylococcal isolates belonging to four *Staphylococcus* species, *S. agnetis* (four isolates), *S. aureus* (four isolates), *S. chromogenes* (eight isolates), and *S. simulans* (eight isolates) were used (Table S1). All isolates originate from bovine clinical or subclinical mastitis and were isolated in quarter milk samples collected from 24 different cows on 22 commercial Finnish dairy herds during a mastitis treatment study (Taponen et al., 2006). When the isolates originated from the same herd, they were of different *Staphylococcus* species and isolated from different cows. In each species, half of the isolates selected for this study were from mastitis cases with mild to moderate clinical signs (clinical mastitis) and half from mastitis cases without clinical signs (subclinical mastitis) (Table S1).

Staphylococci were cultured aerobically on Müller-Hinton (LabM, LAB039) or Trypticase Soy Agar supplemented with 5% bovine blood (bovine blood agar) (Tammer-Tutkan Maljat, 3132) at +37 °C. Hemolysis on bovine blood agar was assessed after 23 h and 42 h at +37 °C. Hemolysis was classified as complete (clear zone around the colonies), incomplete (broad zone of incompletely lysed erythrocytes around the colonies), double (a clear inner zone and a broad outer zone with incomplete hemolysis around the colonies), or non-hemolytic. The strains were maintained at −80 °C in Müller-Hinton broth containing 8.7% (vol/vol) glycerol. One colony from a bovine blood agar culture of each isolate was transferred into tubes with 5 mL Müller-Hinton and incubated overnight at +37 °C. DNA isolation was performed using Easy-DNA™ Kit for genomic DNA isolation (Invitrogen Life Technologies, Carlsbad, CA, USA).

### Genome sequencing and annotation

Genomes of the 20 NAS isolates were sequenced at the Institute of Biotechnology (University of Helsinki, Finland) using next-generation sequencing platforms. The genomes of the four *S. aureus* isolates were sequenced in a previous study (Kant et al., 2015). Genomic DNA (3 µg in 100 µL) was fragmented in a microTube using Covaris S2 (Covaris Inc., Woburn, MA, USA). Half volume (50 µL) was size selected to a mean peak the size of 1.2 kb using magnetic carboxybeads (Lundin et al., 2010). After size selection, the fragment ends were repaired and ligated to 454 Y-adapter as described in the manufacturers protocol (Roche/454 Life Sciences, Branford, CT, USA). The libraries were processed in emulsion PCR and run on a Genome Sequencer FLX+ and the obtained sequences were assembled using the GS Assembler (Roche/454 Life Sciences, Branford, CT, USA).

These 20 newly-sequenced genomes were deposited in GenBank under the accession numbers presented in Table S2. The raw data was deposited to the NCBI Sequence Read Archive (SRA) database under accession number SRP133811 (Table S2). For the annotation process, assembled DNA sequences of the new draft genomes from the 20 isolates were run through an automatic annotation pipeline via RAST (Rapid Annotation using Subsystem Technology) (Aziz et al., 2008), followed by manual curation in some cases.

### Orthologous gene prediction and genome sequence comparison

The EDGAR software platform (Blom et al., 2009), in combination with in-house scripts, was used for identifying the orthologous genes among the 24 genome sequences. To estimate orthologous genes an all-against-all comparison of the genes of all genomes was performed using blastp (Altschul et al., 1997) with the standard scoring matrix BLOSUM62 and an initial *E*-value cut-off of 1e^−05^. The bit score of every blast hit was set into proportion to the best bit score possible, the bit score of a hit of the query gene against itself. This resulted in a score ratio value (SRV) between 0 and 100 that reflected the quality of the hit much better than the raw blast bit score (Lerat, Daubin & Moran, 2003).

Two genes were considered orthologous if a reciprocal best blast hit existed between them, and both hits had an SRV >32. The SRV threshold is calculated from distribution of blast hits between analyzed sequences as described in the supplement of Blom et al. (2009). Based on this orthology criterion, the core genome was calculated as the set of genes that had orthologous genes in all other analyzed strains. The group-wise comparisons were also calculated based on this orthology threshold. A core genome was calculated for the species-specific groups as well as the NAS group, created based on phylogeny and origin. Subsequently, all genes were filtered out of groups having an orthologue in any strain outside the subset.

The pan-genome was calculated as the set of all unique genes of a set of genomes. All genes of one reference genome were considered the basic set for the calculation. Subsequently, the genes of a second genome were compared with this set, and all genes in the second genome that had no orthologous gene in the starting gene set were added to this set. This process was iteratively repeated for all genomes of the compared set, resulting in the pan-genome.

### Identification of the putative virulence factors in the genomes

The Virulence Factor Database (Chen et al., 2016) and the most recent research on the putative virulence factors of *Staphylococcus* were used as guidelines when choosing the putative virulence factors to be sought. The Virulence Factor Database is mostly based on experimentally validated virulence factors. In this database the virulence factors of staphylococci originate mainly from *S. aureus* and *S. epidermidis*. Altogether 163 genes encoding putative virulence factors, divided into 6 classes, were sought in the genomes of the staphylococcal isolates. We used two methods, utilizing either annotated pan-genome or genomes of each isolate to identify the putative virulence factors.

In the first method, both protein and gene names, including the known synonyms, were used as keywords when conducting the searches from the annotated pan-genome sequences, which are based on BLASTP analysis as described in paragraph Orthologous gene prediction and genome sequence comparison in Materials and methods. All the putative virulence factors showing close sequence identity in the pan-genome comparison but discrepancies in the annotated names, as well as about 50 proteins automatically annotated as hypothetical ones but showing homology with the putative virulence factors in pan-genome comparison were manually checked. This was done by aligning and analyzing them in detail with BLAST (NCBI database) and, when available, comparing with reviewed Uniprot (UniProt knowledgebase, http://www.uniprot.org) sequences. The cut-off values used were same as used in the automatic annotation (>40% sequence identity, >50% query coverage). The CLUSTAL omega program (Sievers et al., 2011) was used for multiple protein sequence alignments when needed. Discrepancies observed between the automatic annotation and pan-genome comparison were corrected based on the results of the aforementioned work when feasible.

The second method was based on blasting (BLASTn Megablast, NCBI database) relevant *Staphylococcus* reference sequences, retrieved from GenBank and originating from reviewed protein sequences in UniProt, against the whole genome sequences of our isolates. This method was used for putative virulence factors that were missing at least from one isolate after the search with the first method. If an alignment with at approximately 90% sequence identity and with 90% query coverage was detected, the gene was considered to be present. If a relevant UniProt derived reference sequence was not available for the virulence factor in question, this analysis was not performed.

To compare the virulence factors identified from our 24 *Staphylococcus* isolates, we performed virulence factor searches on publicly available annotated genome sequences of *S. agnetis*, *S. chromogenes*, and *S. simulans* of veterinary origin using the same protein and gene names as keywords as for our own 24 isolates. The accession numbers for these sequences were: JPRT00000000.1 (*S. agnetis* of bovine origin) (Calcutt et al., 2014), PRJNA246192 (BioSample accession number SAMN02743857) (*S. agnetis* strain 908 of broiler origin) (Al-Rubaye et al., 2015), JMJF01000001 (*S. chromogenes* strain Mu 970 of bovine origin) (Fry et al., 2014a), and AXDY01000000 (*S. simulans* UMC-CNS-990 of bovine origin) (Calcutt et al., 2013). For *S. agnetis* strain 908 the putative virulence factors were searched from the supporting information provided in Al-Rubaye et al. (2015).

### Prediction of putatively secreted virulence factors and phylogenetic analysis

For proteins detected by automatic annotation, the secretome of classically secreted or exported proteins was constructed based on the SignalP 4.1 program (SignalP 4.1 Server, http://www.cbs.dtu.dk/services/SignalP) using default settings for Gram-positive bacteria (Petersen et al., 2011). After that, the predicted cellular location of putative virulence factors was compared to their relevant Uniprot reference proteins’ counterparts.

To determine the evolutionary relationships among the various strains of the 24 genomes, we constructed two phylogenetic trees. A phylogenomic tree based on the core-genome was constructed using previously described approaches (Kant et al., 2011). Concisely, a tree was calculated using a slightly adapted version of the pipeline proposed by Zbodnov & Bork (2007), in which predicted protein homologies were used to identify possible genes in each of the different genomes. Here, multiple genome alignments of mutually conserved orthologous genes from the core genome were produced with MUSCLE (Edgar, 2004). After the blocks of multiple alignments were concatenated, any gaps or misaligned regions were then removed with GBLOCKS (Talavera & Castresana, 2007). Phylogenies based on these alignment data were generated using the neighbor-joining algorithm in the PHYLIP package (Felsenstein, 2005). Bionumerics 5.10 (Applied Maths, Sint-Martens-Latem, Belgium) was used to create a phylogenetic tree based on the *rpoB* gene.

### Association between presence of the putative virulence genes and type of mastitis

The possible association between presence of the putative virulence genes and the type of mastitis was evaluated both by visual inspection and statistical analysis. The low number of isolates in each staphylococcal species did not allow a species-specific statistical analysis but the analysis was performed for all NAS species pooled together. Logistic regression was used to study the possible effects of single virulence genes or virulence gene combinations on the type of mastitis. The type of mastitis (clinical/subclinical) was the dependent variable and the virulence genes or gene groups were categorical covariates.

### Urease activity assay

Urease activity of the *Staphylococcus* isolates was determined by inoculating bacterial cells from an overnight bovine blood agar plate onto urea agar slants (Tammer-Tutkan Maljat, Tampere, Finland). The color of the media was examined after an overnight incubation at +37 °C, as well as after two and five days of incubation. Positive urease activity was detected as the formation of a bright-pink color in the media. A color change throughout the urea agar slant was regarded as a positive reaction and a color change only along the slant was regarded as a weak positive reaction.

### Nuclease activity assay

Nuclease activity of the *Staphylococcus* isolates was determined with two different methods, both utilizing commercial methyl-green DNA-plates (Tammer-Tutkan Maljat, Tampere, Finland). The heat-stable nuclease activity was essentially determined as previously described (Madison & Baselski, 1983), with some modifications. For the assay, the isolates were grown in Bacto™ brain-heart infusion broth (BD, 237400; Thermo Fisher Scientific, Waltham, MA, USA) overnight, after which 1 mL of the broth was heat treated in a boiling water bath for 15 min. The methyl-green DNA-plates inoculated with these cells were incubated at +37 °C and the reactions were observed at 24 h. A positive test showed a clear colorless zone around the well cut into the DNA plate, whereas in a negative result the indicator color did not disappear. The deoxyribonuclease (DNase) activity was determined by inoculating bacterial cells from an overnight bovine blood agar plate as streaks on methyl-green DNA plates. Plates were incubated at +37 °C and examined for a positive or a negative reaction around the streak as in the heat-stable nuclease assay at 48 h and 72 h.

### Coagulase assay

For the free coagulase assay two to four colonies from overnight bovine blood agar cultures were mixed with 0.5 mL of rabbit plasma (BBL Coagulase Plasma Rabbit, Becton Dickinson and Company, 240661; Thermo Fisher Scientific, Waltham, MA, USA) in a test tube and examined for clot formation after 1, 2, 3, 4, and 24 h at 37 °C. As the positive control, *S. aureus* type strain DSM 20231^T^ was used, and *S. hyicus* type strain 20459^T^ served as the negative control.

## Results

### General features of the genomes of 24 bovine related *Staphylococcus* isolates

The general features of the 20 new NAS genomes as well as our previously published four *S. aureus* genomes included in this study for comparison are presented in Table S2. The genome size was slightly larger in *S. aureus* (average 2.7 Mbps) and *S. simulans* isolates (average 2.6 Mbps) than in *S. chromogenes* (2.29 Mbps) and *S. agnetis* isolates (average 2.46 Mbps). The numbers of predicted protein-encoding open reading-frames (ORFs) in the 24 isolates varied from 2,116 (*S. chromogenes* 46) to 2,561 (*S. simulans* 15). The total GC content ranged from 32.6 to 37.3% with *S. aureus* isolates having a somewhat lower GC content (32.6%) than the NAS species (average for all the 20 isolates 36%).

### Core and pan-genome of 24 bovine related *Staphylococcus* isolates

**Figure 1:**
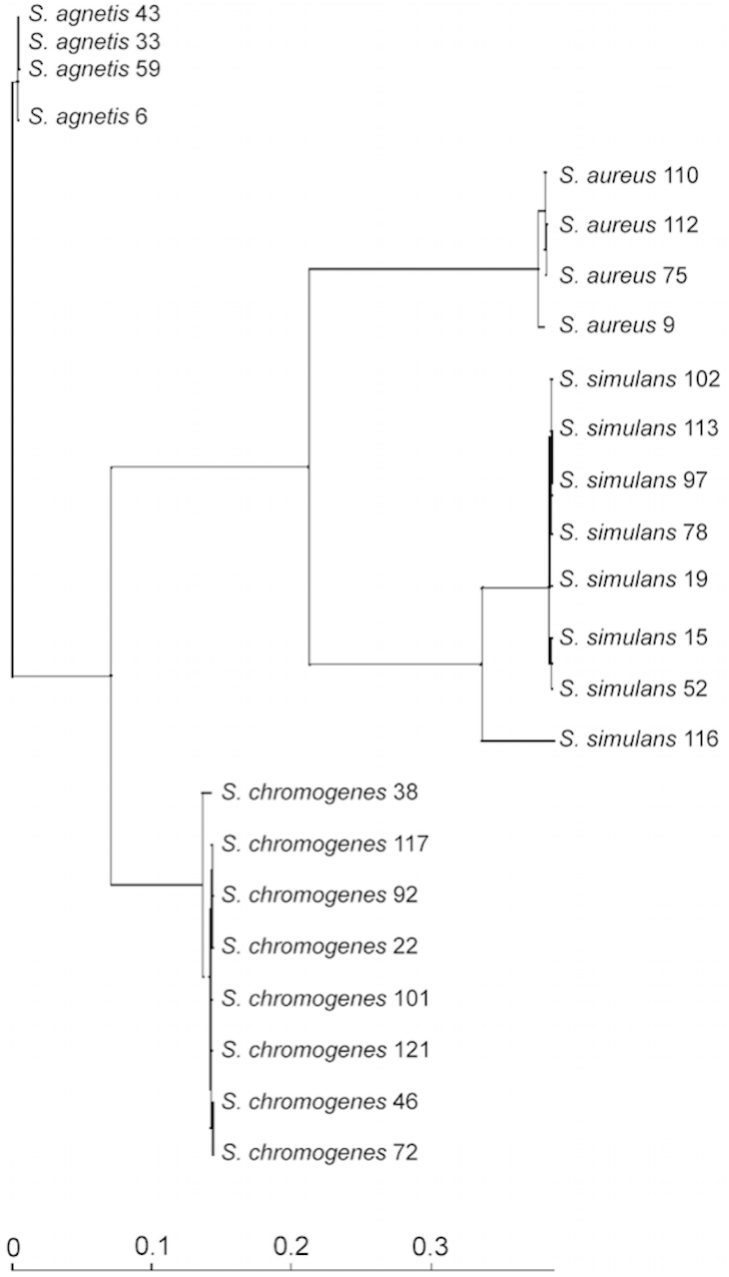
Phylogenetic tree based on the core-genomes of the four *Staphylococcus* species.

**Figure 2:**
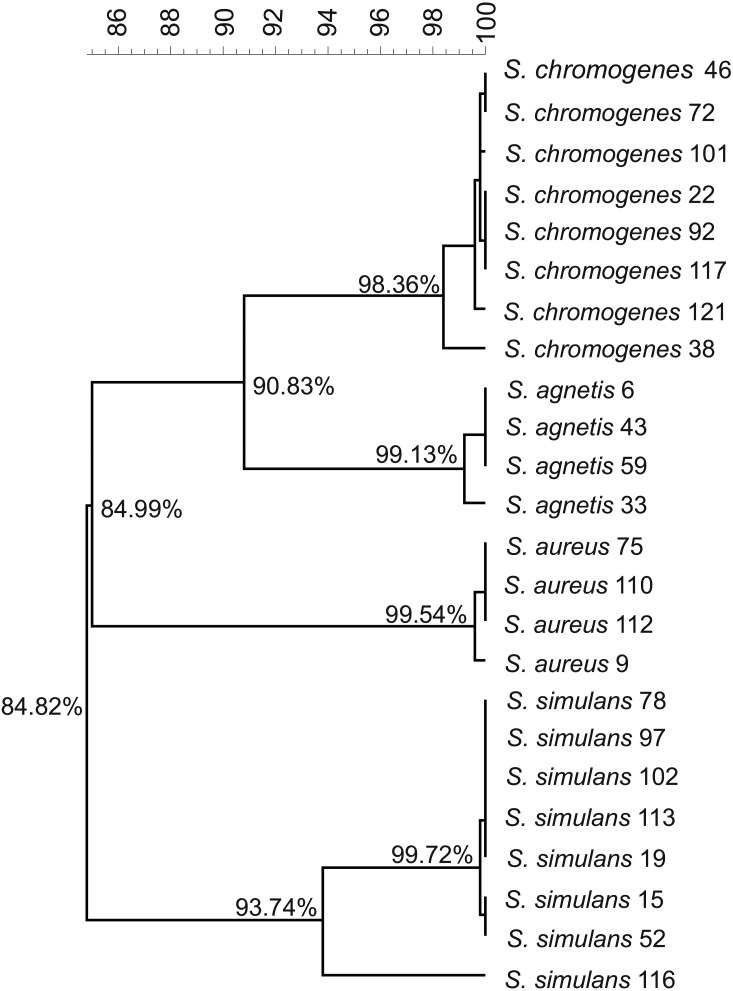
Phylogenetic tree based on the *rpoB.*-gene sequences of all the 24 *Staphylococcus* isolates.

We used the genome sequences of 20 NAS isolates described in this study as well as our previously sequenced four *S. aureus* mastitis isolates (Kant et al., 2015), for comparison, to construct the group and species-specific pan-genomes. As a group, these 24 *Staphylococcus* isolates yielded a pan-genome size of 5,618 genes, of which only 25% (1,430 genes) formed the core-genome, revealing a rather high inter-species diversity. The size of the species-specific pan-genomes of *S. aureus*, *S. agnetis*, *S. chromogens*, and *S. simulans* varied from 2,794 to 3,222 genes, of which 64–80% constituted the core genomes (Table 1). *S. simulans* was identified as having the smallest core-genome (64% of the pan-genome genes) whereas the largest core-genome was observed in *S. aureus* (80% of the pan-genome genes). Figure 1 shows a phylogenetic tree based on the core-genomes of these four *Staphylococcus* species and, for comparison, Fig. 2 shows a phylogenetic tree based on the *rpoB*-gene sequences of all the 24 isolates. Of these four species, *S. agnetis* and *S. chromogenes* are the closest relatives and the similarity between their *rpoB* sequence clusters is 90.83% (Fig. 2).

**Table 1:** Core and pan-genome analysis data on 24 *Staphylococcus* isolates used in the study^a^.

	All	NAS	*S. aureus*	*S. agnetis*	*S. chromogenes*	*S. simulans*
Pan-genome	5,618	4,782	2,815	2,794	2,800	3,222
Core genome	1,430 (25%)	1,521 (32%)	2,254 (80%)	2,128 (76%)	1,955 (70%)	2,055 (64%)
Species specific genes	NR^b^	79	337	115	50	259

**Notes.**

a The number of predicted protein-coding sequences (CDS) are shown and for the core genome CDS the ratio to the pan-genome CDS (% in parenthesis).

b NR, not recorded.

Based on the sequence similarity analysis, all species possessed genes that were absent from the other *Staphylococcus* species (Table 1). In *S. aureus*, 337 such genes were present while the corresponding number for *S. agnetis*, *S. chromogenes*, and *S. simulans* were 115, 50, and 259. In NAS, about 30 to 45% of these genes were annotated as hypothetical proteins in contrast to 5% in *S. aureus*.

### Putative virulence factors of 24 bovine related *Staphylococcus* isolates

The *in silico* identification of the putative virulence genes and the *in vitro* detection of hemolysin, urease, nuclease, and coagulase activity were performed on all isolates. The *in silico* results of each *Staphylococcus* species are listed in Tables 2–9. In addition, the *in silico* results of each *Staphylococcus* isolate are shown in Table S3. The putative virulence factors were classified into six functional groups: toxins, host immune evasion, exoenzymes, adherence, regulatory genes, and miscellaneous virulence-related genes (described below).

**Table 2:** Presence of toxin genes in the 24 *Staphylococcus* isolates of this study.

Virulence factor	Related genes^a^	*S. aureus* (*n* = 4)	*S. agnetis* (*n* = 4)	*S. chromogenes* (*n* = 8)	*S. simulans* (*n* = 8)
***Hemolysins***					
Alpha-hemolysin	*hly*	100% (4)	0	0	0
Phospholipase C (Beta-hemolysin, beta-toxin, sphingomyelinase)	*hlb*	100% (4)	100% (4)	100% (8)	12.5% (1)
Delta-hemolysin (Delta-lysin/ ta-toxin)	*hld*	100% (4)	0	0	0
Hemolysin III	N/A	100% (4)	100% (4)	100% (8)	100% (8)
***Bicomponent leukotoxins***					
Gamma-hemolysin components A, B, and C	*hlgA*	100% (4)	0	0	0
	*hlgC*	100% (4)	0	0	0
	*hlgB*	100% (4)	0	0	0
Panton-Valentine leukocidin (PVL)	*lukS-PV*	100% (4)	0	0	0
	*lukF-PV*	100% (4)	0	0	0
Leukocidin LukED	*lukD*^b^	100% (4)	0	0	12.5% (1)
	*lukE*	100% (4)	0	0	0
***Exfoliative toxins****(epidermolytic toxins)*					
Exfoliative toxin A	*eta*^b^	0	75% (3)	0	0

**Notes.**

a N/A, not assigned/not available.

b Discrepancies observed in combined automatic annotation and pan-genome data; corrected using BLAST analysis.

### Toxins

We identified several predicted virulence factors associated with toxin production. In general, genes encoding different toxins were common in *S. aureus* genomes, but infrequent in the NAS genomes (Tables 2 and 3).

**Table 3:** Presence of staphylococcal superantigen and superantigen-like protein genes in the 24 *Staphylococcus* isolates of this study.

Virulence factor	Related genes^a^	*S. aureus* (*n* = 4)	*S. agnetis* (*n* = 4)	*S. chromogenes* (*n* = 8)	*S. simulans* (*n* = 8)
***Staphylococcal superantigens****(Sags)*					
Toxic shock syndrome toxin-1 (TSST-1)	*tst*	25% (1)	0	0	0
***Enterotoxins and enterotoxin-like toxins***					
Enterotoxin B	*entB (seb)*	25% (1)	0	0	0
Enterotoxin C	*entC (sec)*	25% (1)	0	0	0
Enterotoxin K	*entK (sek)*	100% (4)	0	0	0
Extracellular enterotoxin L	*sel*	25% (1)	0	0	0
Enterotoxin family protein	N/A	100% (4)	0	0	0
Enterotoxin, phage associated	N/A	100% (4)	0	0	0
***Staphylococcal superantigen-like (SSL) proteins***					
Exotoxin	Un-numbered^b^	25% (1)	100% (4)	12.5% (1)	0
	*set1*	100% (4)	0	0	0
	*set4*	100% (4)	0	0	0
	*set6*^b^	100% (4)	0	0	0
	*set7*^b^	100% (4)	100% (4)	100% (8)	0
	*set8*^b^	100% (4)	0	0	0
	*set10*	100% (4)	0	0	0
	*set12*^b^	50% (2)	0	0	0
	*set13*	100% (4)	0	0	0
	*set15*^b^	100% (4)	0	0	0
Streptococcal pyrogenic exotoxin G	*speG*	100% (4)	0	0	0

**Notes.**

a N/A, not assigned/not available.

b Discrepancies observed in combined automatic annotation and pan-genome data.

#### Hemolysins

Alpha- and delta-hemolysin orthologues were only present in *S. aureus* isolates (Table 2, Table S3). Phospholipase C (beta-hemolysin) orthologues were identified in all *S. aureus*, *S. agnetis*, and *S. chromogenes* isolates, whereas in *S. simulans* this hemolysin was only detected in one isolate (Table 2). A hemolysin-3 orthologue was detected in all 24 isolates.

To determine whether the genomic hemolysin gene patterns correlate with the *in vitro* hemolysis, the *in vitro* hemolysis patterns were observed after 23 h and 42 h of incubation on bovine blood agar. In all *S. aureus* isolates, double hemolysis was detected after 23 h of incubation (Table S4). The patterns of hemolysis differed between the three NAS species. In all isolates of *S. simulans* and all but one *S. chromogenes* a complete hemolysis was detected, but the reaction was sometimes, especially among *S. chromogenes* isolates, clearly visible only after 42 h of incubation. The *S. agnetis* isolates showed no hemolysis.

#### Bicomponent leukotoxins

Genes encoding gamma-hemolysin component A, B, and C orthologues, as well as Panton-Valentine leucocidin F and S orthologues, were present in all four *S. aureus* isolates but none of the NAS isolates (Table 2, Table S3). The genes *lukS-PV* and *lukF-PV* coding for the bicomponent leukotoxin Panton-Valentine leukocidin were also found in all *S. aureus* isolates. A *lukD* orthologue, but not a *lukE* orthologue, was also identified in one *S. simulans* isolate. The genes *lukM* and *lukF’-PV*, coding for the bicomponent leukocidin LukM/F’, were not detected in any of the isolates.

#### Exfoliative toxins

The gene *eta* encoding exfoliative toxin type A was detected in three of the four *S. agnetis* isolates (75%), but not in other species. The genes *etb*—*etd* encoding exfoliative toxin types B, C and D were not identified in any of the isolates (Table 2).

#### Staphylococcal superantigens (Sags) and superantigen-like (SSL) proteins

None the NAS isolates carried any of the genes coding for staphylococcal superantigens (Table 3), whereas orthologues for genes coding for enterotoxin K (*entK*), enterotoxin family protein, and phage associated enterotoxin were detected in all *S. aureus* isolates. The *tst* gene coding for toxic shock syndrome toxin, the *entB* and *entC* genes coding for enterotoxin types B and C, and the *sel* gene encoding extracellular enterotoxin L were concurrently detected in a single *S. aureus* isolate.

*S. aureus* isolates harbored several staphylococcal superantigen-like protein encoding genes, as a large proportion of the exotoxin genes *set 1-15* were detected in them (Table 3). The exotoxin genes *set1, 4, 6, 7, 8, 10, 13*, and *15* were found in all the *S. aureus* isolates, and the gene *set12* in two out of four isolates. The gene *set7* was the only *set*-gene found in NAS isolates, occurring in all of *S. agnetis* and *S. chromogenes* isolates*.* In addition, unnumbered exotoxin gene orthologues were identified in one *S. aureus*, one *S. chromogenes*, and all four *S. agnetis* isolates. The *S. simulans* isolates did not carry any exotoxin genes. The *speG* gene coding for streptococcal pyrogenic exotoxin G was found in all four *S. aureus* isolates but no NAS isolates.

### Host immune evasion

#### Capsule type specific genes

Several genes coding for either capsule type 5 or 8 were detected in *S. aureus* and NAS isolates (Table 4). Most (75%) of the *S. aureus* isolates harbored a complete set of four type specific genes coding for capsule type 8, while the majority (75%) of the *S. agnetis* isolates harbored a complete set of four type specific genes coding for capsule type 5, together with one *S. aureus* (25%) isolate and one (13%) *S. chromogenes* isolate. The rest of the studied *S. chromogenes* isolates did not harbor any capsule genes studied. In one *S. agnetis* isolate (25%), three of the orthologues associated with capsule type 5 were present. In every *S. simulans* isolate, only one orthologue, associated with capsule type 8, was present.

**Table 4:** Presence of host immune evasion genes in the 24 *Staphylococcus* isolates of this study.

Virulence factor	Related genes	*S. aureus* (*n* = 4)	*S. agnetis* (*n* = 4)	*S. chromogenes* (*n* = 8)	*S. simulans* (*n* = 8)
Capsular biosynthesis proteins^b^	*cap5H*	25% (1)	100% (4)	12.5% (1)	0
	*cap5I*	25% (1)	100% (4)	12.5% (1)	0
	*cap5J*	25% (1)	75% (3)	12.5% (1)	0
	*cap5K*	25% (1)	100% (4)	12.5% (1)	0
	*cap8H*	75% (3)	0	0	0
	*cap8I*	75% (3)	0	0	87.5% (7)
	*cap8J*	75% (3)	0	0	0
	*cap8K*	75% (3)	0	0	12.5% (1)
Fibrinogen-binding protein	*fib* (*efb*)	100% (4)	25% (1)	0	0
Immunoglobulin-binding protein sbi^a^	*sbi*	100% (4)	25% (1)	12.5% (1)	0
Phosphatidylglycerol lysyltransferase^b^ (Multiple peptide resistance factor)	*mprF* (*fmtC*)	100% (4)	100% (4)	100% (8)	75% (6)

**Notes.**

a Discrepancies observed in combined automatic annotation and pan-genome data; corrected using BLAST analysis.

b Discrepancies observed in combined automatic annotation and pan-genome data.

#### Other genes related to the immune evasion

The gene *fib*, coding for fibrinogen binding protein, was present in all *S. aureus* isolates and one *S. agnetis* isolate (Table 4). The gene *sbi*, coding for IgG-binding protein Sbi, was present in all *S. aureus* isolates, one *S. agnetis* isolate (the same isolate carrying also *fib*), and one *S. chromogenes* isolate. The gene *mprF*, coding for phosphatidylglycerol lysyltransferase, was present in all the *S. aureus, S. agnetis* and *S. chromogenes* isolates, and most (75%) of the *S. simulans* isolates. Orthologues for chemotaxis inhibiting protein CHIPS and for staphylococcal complement inhibitor were not detected in any of the isolates.

### Exoenzymes

#### Iron uptake

All *S. aureus* isolates possessed a complete set of all the nine iron-regulated surface (*isd*) locus genes *isdA*-*isdI* (Table 5). In the NAS species, a large variety of *isdA-isdI* genes were observed (Table 5). The *S. agnetis* and *S. chromogenes* isolates more commonly harbored only the *isdI* gene of this set of putative virulence factors, whereas most *S. simulans* isolates also possessed the genes *isdC, isdE, isdF*, and *isdG*. A sortase A orthologue was present in all *Staphylococcus* isolates, whereas sortase B orthologues were found in all *S. aureus* isolates and in the majority (88%) of *S. simulans* isolates (Table 5).

**Table 5:** Presence of genes related with iron uptake in the 24 *Staphylococcus* isolates of this study.

Virulence factor	Related genes	*S. aureus* (*n* = 4)	*S. agnetis* (*n* = 4)	*S. chromogenes* (*n* = 8)	*S. simulans* (*n* = 8)
Iron-regulated surface determinant proteins	*isdA*^b^	100% (4)	0	37.5% (3)	25% (2)
	*isdB* (*sasJ*)	100% (4)	0	0	0
	*isdC*^b^	100% (4)	0	0	87.5% (7)
	*isdH* (*sasI*)	100% (4)	0	0	0
Heme transporter IsdDEF, membrane component IsdD	*isdD*	100% (4)	0	0	0
High-affinity heme uptake system protein IsdE	*isdE*	100% (4)	0	0	87.5% (7)
Probable heme-iron transport system permease protein IsdF	*isdF*	100% (4)	0	0	75% (6)
Heme oxygenases (staphylobilin-producing)	*isdG*^a^	100% (4)	0	0	100 (8)
	*isdI*^b^	100% (4)	100% (4)	100% (8)	87.5% (7)
Sortases A and B	*srtA*	100% (4)	100% (4)	100% (8)	100% (8)
	*srtB*	100% (4)	0	0	87.5% (7)

**Notes.**

a Discrepancies observed in combined automatic annotation and pan-genome data; corrected using BLAST analysis.

b Discrepancies observed in combined automatic annotation and pan-genome data.

#### Other exoenzymes

Catalase orthologues, as expected, were found and thermonuclease orthologues identified in all *Staphylococcus* isolates (Table 6). In the *in vitro* heat-stable nuclease assay, all the *S.  aureus*, *S. chromogenes*, and *S. agnetis* isolates gave a positive reaction, whereas all the *S.  simulans* isolates gave a negative result (Table S5). The results of the deoxyribonuclease assay were identical with results of the heat-stable nuclease assay (Table S5).

**Table 6:** Presence of exoenzyme genes in the 24 *Staphylococcus* isolates of this study.

Virulence factor	Related genes	*S. aureus* (*n* = 4)	*S. agnetis* (*n* = 4)	*S. chromogenes* (*n* = 8)	*S. simulans* (*n* = 8)
Catalase	*katA*	100% (4)	100% (4)	100% (8)	100% (8)
Staphylocoagulase	*coa*	100% (4)	100% (4)	0	0
Glyseraldehyde 3-phosphate dehydrogenase	*gapA1, gapA2*	100% (4)	100% (4)	100% (8)	100% (8)
Hyaluronate lyase (Hyaluronidase)	*hysA*	100% (4)	100% (4)	0	0
von Willebrand factor binding protein	*vwb*	100% (4)	100% (4)	100% (8)	0
Staphylokinase	*sak*	100% (4)	0	0	0
Thermonuclease	*nuc*	100% (4)	100% (4)	100% (8)	100% (8)
Zinc metalloproteinase aureolysin	*aur*	100% (4)	100% (4)	100% (8)	0
Glutamyl endopeptidase (V8 protease)	*sspA*	100% (4)	0	0	0
Staphopain A and B (Cysteine proteinase A and B)	*sspP* (*scpA*)	100% (4)	100% (4)	0	87.5% (7)
	*sspB*	100% (4)	0	0	0
Staphostatin B (Cysteine protease inhibitor B)	*sspC*	100% (4)	0	0	0

**Notes.**

a Discrepancies observed in combined automatic annotation and pan-genome data; corrected using BLAST analysis.

b Discrepancies observed in combined automatic annotation and pan-genome data.

Staphylocoagulase and hyaluronate lyase orthologues were identified in all *S. aureus* and *S. agnetis* isolates, but not *S. simulans* and *S.  chromogenes* isolates (Table 6). In the *in vitro* tube coagulase assay detecting both staphylocoagulase and clumping factor, all the *S. aureus* isolates were positive in 2 h, whereas only one of the *S. agnetis* isolates gave a positive reaction within 24 h (Table S5).

The gene predicted to encode glutamyl endopeptidase was identified in all of the *S. aureus* isolates (Table 6). The von Willebrand factor binding protein and zinc metalloproteinase aureolysin orthologues were not identified in any of the *S. simulans* isolates, but were prevalent in all other *Staphylococcus* isolates. Orthologues for glyceraldehyde-3-phosphate dehydrogenase were present in all isolates. All the *S. aureus* isolates had staphopain A and B orthologues as well as staphostatin B orthologues, whereas only orthologues for staphopain A were identified in *S. agnetis* and *S. simulans* (Table 6). Staphylokinase orthologues were not found in any isolates.

### MSCRAMMs

Numerous orthologues for genes encoding different microbial surface components recognizing adhesive matrix molecules (MSCRAMM) were detected in our isolates (Table 7, Table S3). Orthologues for genes encoding elastin binding protein EbpS, enolase, and fibronectin/fibrinogen-binding protein were present in all isolates. The *spa* gene, encoding immunoglobulin G-binding protein A, was present in all isolates of *S. aureus*, *S. agnetis*, and *S. chromogenes* but not in *S. simulans* isolates. The gene *bap,* encoding biofilm-associated protein, was detected in all *S. agnetis* and one of *S. chromogenes* isolates, but not in any of the *S. aureus* or *S. simulans* isolates. The gene *cna* coding for collagen adhesin was present in all *S. agnetis* isolates, but not isolates of other species. The gene *map*, encoding for extracellular adherence protein of broad specificity Eap/Map, and gene *ebh*, encoding for extracellular matrix-binding protein, were present in all the *S. aureus* isolates, but not in isolates of other species (Table S3).

**Table 7:** Presence of MSCRAMM genes in the 24 *Staphylococcus* isolates of this study.

Virulence factor	**Related genes**	*S. aureus***(*n* = 4)**	*S. agnetis***(*n* = 4)**	*S. chromogenes***(*n* = 8)**	*S. simulans***(*n* = 8)**
Collagen adhesin	*cna*	0	100% (4)	0	0
Elastin-binding protein EbpS	*ebpS*	100% (4)	100% (4)	100% (8)	100% (8)
Enolase	*eno*	100% (4)	100% (4)	100% (8)	100% (8)
Extracellular adherence protein of broad specificity Eap/Map	*map*	100% (4)	0	0	0
Fibronectin binding proteins	*fnbA*/*fnbB*^a^	100% (4)	100% (4)	100% (8)	0
Fibronectin/fibrinogen binding protein	*fbe*	100% (4)	100% (4)	100% (8)	100% (8)
Polysaccharide intercellular adhesin proteins (Biofilm operon icaADBC and HTH-type negative transcriptional regulator IcaR)	*icaR*	100% (4)	0	0	87.5% (7)
	*icaA*	100% (4)	0	0	0
	*icaB*	100% (4)	0	0	0
	*icaC*	100% (4)	0	0	100% (8)
	*icaD*	50% (2)	0	0	0
IgG-binding protein A (Staphylococcal protein A)	*spa*^a^	100% (4)	100% (4)	100% (8)	0
Serine-rich adhesin for platelets (S. aureus surface protein A)	*sraP*^b^ (*sasA*)	100% (4)	0	0	37.5% (3)
Cell-wall-anchored proteins SasC, SasD and SasK	*sasC*	100% (4)	0	0	0
	*sasD*	100% (4)	0	0	0
	*sasK*	25% (1)	0	0	0
Predicted cell-wall-anchored protein sasF	*sasF*^a^	100% (4)	100% (4)	100% (8)	100% (8)
Surface protein G	*sasG*	25% (1)	0	0	0
Surface protein SasH	*sasH*	100% (4)	100% (4)	100% (8)	100% (8)
Biofilm-associated protein	*bap*	0	100% (4)	12.5% (1)	0
*Sdr-family*					
Clumping factor A	*clfA*	75% (3)	0	0	0
Clumping factor B	*clfB*	100% (4)	0	0	0
Serine-aspartate repeat-containing proteins C-E and I	*sdrC*^b^	100% (4)	0	100% (8)	12.5% (1)
	*sdrD*^b^	0	0	0	50% (4)
	*sdrE*^b^	75% (3)	0	0	37.5% (3)
	*sdrI*^a^	0	75% (3)	12.5% (1)	0

**Notes.**

a Discrepancies observed in combined automatic annotation and pan-genome data; corrected using BLAST analysis.

b Discrepancies observed in combined automatic annotation and pan-genome data.

The genes *fnbA* and/or *fnbB* orthologues, encoding fibronectin binding proteins FnbA and FnbB, were present in all of *S. aureus*, *S. agnetis*, and *S. chromogenes* isolates, but not in any of the *S. simulans* isolates (Table 7, Table S3). As several of these sequences did not overlap the variable region A of proteins FnbA and FnbB (Murai et al., 2016), which is the sole region able to differentiate *fnbA* and *fnbB* orthologues, all the *fnbA/fnbB* orthologues were counted together (Table 7).

Every *S. aureus* isolate carried orthologues for all the genes of the *ica* (intercellular adhesion) cluster ABCD and a gene encoding HTH-type negative transcriptional regulator IcaR. None of these genes were detected in *S. agnetis* and *S. chromogenes* (Table 7). Of the *S. simulans* isolates, all carried *icaC* and most (88%) *icaR*.

Of the *sraP* (also known as *sasA*), *sas* orthologues encoding staphylococcal surface proteins, *sasF* and *sasH* were most prevalent, as all isolates harbored both of them (Table 7). The *sraP* gene was present in all *S. aureus* and 38% of *S. simulans* isolates. The genes *sasC* and *sasD* were present in all *S. aureus* isolates. In addition, a single *S. aureus* isolate was detected to harbor both the *sasG* and *sasK* orthologues, whereas all of these genes were missing from the other species. The *sasB* orthologues were not detected in any isolate.

In almost all (88%) isolates at least one orthologue for serine-aspartate (SD) dipeptide repeat (Sdr) family proteins was present. The bone sialoprotein binding protein encoding orthologue *bbp* and *clfA* and *clfB* orthologues, encoding clumping factor A or B, were only identified in *S. aureus* isolates (Table 7). All *S. aureus* isolates had the *bbp* and *clfB* genes and the majority (75%) also had *clfA* (Table S3). None of the *S. agnetis* isolates harbored the *sdrC*-*sdrE* orthologues predicted to encode Ser-Asp repeat-containing proteins C–E, but most isolates of the other *Staphylococcus* species possessed at least one of these (Table 7). The gene encoding SdrI surface protein was found in almost (75%) all *S. agnetis* isolates and in one (13%) *S. chromogenes* isolate (Table  7). Orthologues for serine-aspartate repeat-containing proteins F–H were not found in any isolate.

### Regulatory genes

Of the genes belonging to the accessory gene regulator quorum-sensing system, *agrA*, *agrB*, and *agrC* orthologues were found in all isolates, but *agrD* was only identified in the *S. aureus* isolates (Table 8). Gene orthologues encoding histidine protein kinase SaeS and response regulator SaeR, belonging to the two component regulatory system SaeR/SaeS, were present in all *S. aureus* and *S. simulans* isolates.

**Table 8:** Presence of regulatory genes in the 24 *Staphylococcus* isolates of this study.

Virulence factor	**Related genes**	*S. aureus***(*n* = 4)**	*S. agnetis***(*n* = 4)**	*S. chromogenes***(*n* = 8)**	*S. simulans***(*n* = 8)**
Accessory gene regulator proteins	*agrA*^a^	100% (4)	100% (4)	100% (8)	100% (8)
	*agrB*	100% (4)	100% (4)	100% (8)	100% (8)
	*agrC*^a^	100% (4)	100% (4)	100% (8)	100% (8)
	*agrD*^a^	100% (4)	0	0	0
Response regulator SaeR and Histidine protein kinase SaeS^a^ of two component regulatory system saeR/saeS	*saeR*	100% (4)	0	0	100% (8)
	*saeS*	100% (4)	0	0	100% (8)
*MarR/SlyA-family, Sar-subfamily of transcriptional regulators*					
HTH-type transcriptional regulator rot (Repressor of toxins)	*rot*	100% (4)	100%(4)	100% (8)	0
Transcriptional regulator SarA (Staphylococcal accessory regulator A)	*sarA*^a^	100% (4)	100%(4)	100% (8)	100% (8)
HTH-type transcriptional regulators	*sarR*^a^	100% (4)	100% (4)	100% (8)	100% (8)
	*sarS*^a^	100% (4)	0	0	0
	*sarT*	25% (1)	0	0	0
	*sarU*	25% (1)	0	0	0
	*sarV*	100% (4)	0	0	100% (8)
	*sarX*	100% (4)	0	0	0
	*sarZ*^a^	100% (4)	100% (4)	100% (8)	100% (8)
	*mgrA*^a^	100% (4)	100% (4)	100% (8)	100% (8)

**Notes.**

a Discrepancies observed in combined automatic annotation and pan-genome data; corrected using BLAST analysis.

**Table 9:** Presence of miscellaneous virulence-related genes in the 24 *Staphylococcus* isolates of this study.

Virulence factor	**Related genes**	*S. aureus***(*n* = 4)**	*S. agnetis***(*n* = 4)**	*S. chromogenes***(*n* = 8)**	*S. simulans***(*n* = 8)**
Surface protein (Plasmin-sensitive surface protein)	*pls*^b^	25% (1)	0	62.5% (5)	50% (4)
Urease subunits	*ureA*	100% (4)	100% (4)	100% (8)	100% (8)
	*ureB*	100% (4)	100% (4)	100% (8)	100% (8)
	*ureC*	100% (4)	100% (4)	100% (8)	100% (8)
Urease accessory proteins	*ureD*	100% (4)	100% (4)	100% (8)	100% (8)
	*ureE*	100% (4)	100% (4)	100% (8)	100% (8)
	*ureF*	100% (4)	100% (4)	100% (8)	100% (8)
	*ureG*	100% (4)	100% (4)	100% (8)	100% (8)
Probable transglycosylase IsaA (Immunodominant staphylococcal antigen A)	*isaA*^b^	100% (4)	0	100% (8)	100% (8)
Immunodominant staphylococcal antigen B	*isaB*	100% (4)	100% (4)	100% (8)	0
***Gas vesicle proteins***					
Gas vesicle protein GvpU	*gvpU*^b^	0	100% (4)	12.5% (1)	0
Gas vesicle structural protein GvpJ	*gvpJ*^a^	0	100% (4)	12.5% (1)	87.5% (7)
Gas vesicle structural protein GvpA	*gvpA*^a^	0	100% (4)	12.5% (1)	87.5% (7)
Gas vesicle structural protein GvpS	*gvpS*	0	100% (4)	12.5% (1)	87.5% (7)
Gas vesicle protein GvpK	*gvpK*	0	100% (4)	12.5% (1)	87.5% (7)
Protein GvpL/F (Gas vesicle protein, GvpL/GvpF family)	*gvpF, gvpL*^a^	0	100% (4)	12.5% (1)	87.5% (7)

**Notes.**

a Discrepancies observed in combined automatic annotation and pan-genome data; corrected using BLAST analysis.

b Discrepancies observed in combined automatic annotation and pan-genome data.

Several transcriptional regulator gene orthologues of the Sar-subfamily in MarR/SlyA-family were detected in all isolates (Table 8). Transcriptional regulator SarA and HTH-type transcriptional regulators SarR, SarZ, and MgrA orthologues were found in all isolates. HTH-type transcriptional regulator Rot orthologues were present in all *S. aureus, S. agnetis*, and *S. chromogenes* isolates, but were not identified in any *S. simulans* isolate. The *sarV* orthologues were detected in all *S. aureus* and *S. simulans* isolates, but *sarS* and *sarX* orthologues were only present in *S. aureus* isolates. The *sarT* and *sarU* orthologues were only detected in one *S. aureus* isolate. The *sarY* orthologues were not identified in any isolate.

### Miscellaneous virulence-related genes

The *isaA* orthologue, encoding probable transglycosylase IsaA, also called immunodominant staphylococcal antigen A, was found in every isolate of all species except *S. agnetis* (Table 9), whereas the *isaB* gene, encoding immunodominant antigen B, was found in every isolate of all species except *S. simulans* (Table 9). The gene *pls* coding for surface protein (plasmin-sensitive surface protein) was found in more than half of the *S. chromogenes* (63%), in half of the *S. simulans* isolates, and one *S. aureus* isolate, but no *S. agnetis* isolates (Table 9). Epidermal cell differentiation inhibitor orthologues were not found in any of the isolates.

Urease gene orthologues, *ureABCEFGD*, were found in all isolates (Table 9). In *S. aureus* and *S. simulans* the urease genes were found to be located in a single operon with the orientation *ureABCEFGD* (Table S6). In *S. agnetis* and *S. chromogenes*, however, the urease genes were found to be located in two operons: the genes encoding accessory proteins ureEFGD in one operon and the genes *ureABC* encoding subunit proteins in a second operon (Table S6). The in vitro urease activities of the four *Staphylococcus* species differed. Half of *S. aureus* and all *S. simulans* isolates gave a positive reaction in 24 h. Most of the *S. chromogenes* isolates were also urease positive, but the reaction was typically delayed (75%) and weak (63%). Only one of the *S. agnetis* isolates was urease positive *in vitro*, giving a weak delayed reaction (Table S6).

The genes encoding putative gas vesicle proteins Gvp were most prevalent in *S. agnetis* and *S. simulans* isolates. Only one *S. chromogenes* isolate and no *S. aureus* isolates possessed these genes (Table 9). The *GvpA* gene orthologue encoding the major protein component of the gas vesicle wall (Pfeifer, 2012) was present in all NAS isolates.

### Predicted secretion of the putative virulence factors

We compared the cellular location of putative virulence factors identified from our isolates to their relevant Uniprot reference proteins counterparts according to the signal sequence predictions of the SignalP program. The cellular location of the majority of the virulence factors identified from our *Staphylococcus* isolates was in line with their respective reference sequences retrieved from Uniprot. Almost all of the differences found concerning the cellular location were such that the virulence factors identified from our isolates were not predicted to be secreted despite their counterpart UniProt reference sequences being predicted to be extracellular or located in the cell wall (Table  S3). However, since all the gene sequences were necessarily not complete, it is possible that some of the sequences were lacking the signal sequence thus explaining observed discrepancies. In addition, our secretion prediction was only performed using SignalP, and a part of the putative virulence factors may be secreted with a different route.

### Association between presence of the putative virulence genes and type of mastitis

By visual inspection it was impossible to find any association between the type of mastitis and specific virulence genes, virulence gene profiles, or cumulative number of virulence genes (Table S3). Logistic regression analyses of the pooled NAS data did not either yield statistically significant (*p* < 0.5) effects of any virulence genes or groups of genes on the type of mastitis.

Most (117) of the virulence genes lacked intra-specific variation. They were either present (24 genes) or absent (39 genes) in all of the 24 isolates, or present (54 genes) in all the isolates in some of the species (mostly in *S. aureus*) and then absent in all the isolates of some other species. The rest, 46 genes may, at least in some extend, be associated with clinical (or subclinical) outcome. However, the differences between clinical and subclinical isolates were small. Four genes were present only in clinical isolates, yet all these genes were singletons. In addition, 14 genes were found more frequently within the clinical than subclinical isolates. Four genes, all singletons, were present only in subclinical isolates. In addition, 16 genes were found more frequently in subclinical than clinical isolates.

No association between virulence gene profiles and the type of mastitis were found. Most of the isolates had unique virulence gene profiles. When two isolates in one species shared an identical profile, as within *S. agnetis*, *S. chromogenes* and *S. simulans*, one of the isolates was clinical while the other was subclinical.

The numbers of virulence genes detected in *S. aureus*, *S. agnetis*, *S. chromogenes*, and *S. simulans* were 98–105, 52–53, 37–49, and 38–48, respectively (Table S3). In *S. aureus*, *S. chromogenes* and *S. simulans*, the highest cumulative counts were found only among clinical isolates, while in *S. agnetis* and *S. chromogenes*, the lowest counts were found only among subclinical isolates. However, most of the cumulative counts were shared with both clinical and subclinical isolates within each of the species.

## Discussion

To our knowledge, this is so far the most extensive study of the putative virulence factors of staphylococci originating from bovine mastitis. We found marked differences in genes expected to influence virulence in 24 staphylococcal isolates of species *S. aureus, S. agnetis, S. chromogenes*, and *S. simulans*. As expected, the four *S. aureus* isolates had many more putative virulence gene orthologues than the 20 isolates belonging to the three NAS species. One third of the detected virulence factors occurred only in *S. aureus*. In all *S. aureus* isolates, nearly 100 different putative virulence factors were present compared to 40 to 50 in each NAS isolate. We also compared the presence of putative virulence factors between *S. aureus* and the NAS isolates as one group. None of the virulence factors occurred in all the NAS isolates without occurring in *S. aureus* isolates. Differences also existed between the NAS species as less than half of the detected putative virulence factors occurred in all species. *S. simulans* differed the most from the other two NAS species in that several of the putative virulence genes detected only arose in *S. simulans*. *S. simulans*, however, also lacked many putative virulence genes present in both *S. agnetis* and *S. chromogenes*.

The importance of the role of the different putative virulence factor genes on the clinical characteristics and persistence of bovine mastitis is still not fully understood. Virulence factors are seen as properties (i.e., gene products) that enable a microorganism to establish itself on or within a host of a particular species and enhance its potential to cause disease (Virulence Factor Database). Thus, any property of the microorganism which enhances its’ potential to survive within a host can be seen as a virulence factors. The line between virulence factors and eg. proteins involved in normal metabolism is not as clear as thought earlier. Therefore, the presence of a single virulence factor rarely determines the microorganisms’ capacity to act as a pathogen. In addition, also the expression level of the virulence factors may have an influence on the outcome of the disease (Le Maréchal et al., 2011).

In this study, we did not observe any clear difference in the virulence gene profiles or cumulative number of different virulence genes between the isolates from clinical and subclinical mastitis. Haveri et al. (2005) and Haveri et al. (2007) also found that while the most prevalent pulsotypes were associated with certain types of mastitis (symptoms, persistence, and response to the antimicrobial treatment) (Haveri et al., 2005), such association was not found with any single virulence gene or gene group (e.g., hemolysin genes) or the cumulative number of virulence genes (Haveri et al., 2007). These results indicate that, possibly excluding the most severe peracute *S. aureus* mastitis, similar symptoms can be caused by several different combination of virulence factors rather than by any of them alone. In addition, not only the properties of microbes but also the immune system of the host, the cow, has an important role in the manifestation of the inflammation. All our isolates originated from mastitis with mild to severe clinical signs and, unfortunately, not from peracute clinical mastitis. Non-aureus staphylococci seldom cause severe mastitis, but *S. aureus* is well-known for its ability to cause severe, even peracute mastitis leading to toxemic necrosis of the affected udder quarter and death of the cow (Taponen & Pyörälä, 2009). The interesting question, are such severe symptoms consequences of some specific virulence factors of the bacteria or an extremely strong immune reaction of the cow, remains to be solved.

Several studies have screened the presence of virulence factors, especially *S. aureus* isolates of bovine milk origin, using mostly either conventional PCR or DNA microarray methods. Only a few studies concentrated on NAS, and these studies have mainly focused on detection of genes associated with biofilm formation (Piessens et al., 2012; Simojoki et al., 2012; Tremblay et al., 2013). To gain insight into the virulence profiles of the NAS species we also included all the publicly available annotated genome sequences of *S. agnetis*, *S. chromogenes* and *S. simulans* of veterinary origin and searched the putative virulence factors from these isolates (Calcutt et al., 2013; Calcutt et al., 2014; Fry et al., 2014a; Seixas et al., 2014; Al-Rubaye et al., 2015). Altogether, the virulence gene profiles of these (Table S7) and our own isolates were similar to each other and profiles found in other studies (Akineden et al., 2001; Srinivasan et al., 2006; Thomas et al., 2006; Kalorey et al., 2007; Sung, Lloyd & Lindsay, 2008; Hart, Hart & Roop, 2009; Oliveira et al., 2011; Wilson et al., 2011; Piessens et al., 2012; Simojoki et al., 2012; Tang et al., 2013; Tremblay et al., 2013; Nemeghaire et al., 2014; Salaberry et al., 2015; Snell et al., 2015; Xu et al., 2015; Adkins, Middleton & Fox, 2016; Artursson et al., 2016; Casaes Nunes et al., 2016; Kot et al., 2016; Mello et al., 2016). Some differences existed, however, mostly on the presence/absence of toxins. For example, delta hemolysin is usually produced by nearly 100% of *S. aureus* isolates (Monecke et al., 2007; Da Costa et al., 2014), but in our study the gene for this hemolysin was found in only one. *S. aureus* isolates carried both the alpha hemolysin and phospholipase C genes, which explains their different hemolyses compared to the hemolysis patterns of the NAS *in vitro*. Interestingly, the differences in *in vitro* hemolysis observed between the different NAS species and single isolates within this group cannot be fully explained with the presence/absence of hemolysin genes.

The urease genes, *ureABCEFGD*, occurred in all our isolates, but not in the *S. chromogenes* isolate sequenced by Fry et al. (2014a) (Table S7). In *S. aureus* and *S. simulans* the urease genes were located in a single operon with the orientation *ureABCEFGD*, as previously reported for *S. aureus*, *S. epidermidis*, and *Staphylococcus saprophyticus* (Kuroda et al., 2005). In our *S. agnetis* and *S. chromogenes*, the urease genes were located in two operons. The operon structure of the urease genes could be one putative factor explaining the different *in vitro* urease results of the studied isolates. The location of urease genes in a single operon seems to lead to a strong and fast positive urease reaction, whereas the separation of the urease genes into two operons appears mainly to give either a delayed or negative urease (Table S6). Comparisons between the in vitro urease activities of clinical and subclinical isolates showed no major differences, as both strong and weak delays as well as negative reactions were observed in both isolate types. To our knowledge no studies exist on the role urease plays in *S. aureus* in the pathogenesis of either veterinary or human infections. One can speculate that urease activity is beneficial for a bovine mastitis causing pathogen, as milk urea concentration of high yielding dairy cows is usually substantial, reflecting the blood urea concentration and intense protein metabolism (Huhtanen et al., 2015).

Gas vesicles are intracellular structures composed of proteins and filled with gas providing the bacterial cells with the capacity to buoy in liquid when present in adequate amounts (Pfeifer, 2012). Thus far these structures have not been described in any members of the *Staphylococcus* genus (Pfeifer, 2012; Calcutt et al., 2014), however, genes related to these structures have previously been identified from *S. agnetis* (Calcutt et al., 2014) and *S. simulans* (Calcutt et al., 2013) but to our knowledge not *S. aureus*. In our study, several putative genes for gas vesicle structures were identified in *S. agnetis* and *S. simulans* and in one *S. chromogenes* isolate. In the literature, *S. chromogenes* has not reportedly possessed these genes. When present, the *gvp* genes clustered together in a single locus in the genome, akin to the previously characterized *S. agnetis* and *S. simulans gvp* genes. Whether these structures form *in vivo* and, if so, what their possible function is in a mammary gland during infection requires further studies.

Heterogeneous naming of the virulence factors in the databases (Uniprot, NCBI) complicated this study. The same virulence factors were often found in the databases under multiple names, hampering the identification of the detected, putative virulence factors, especially if no reviewed reference sequences in the Uniprot database were available. We resolved the misnomers to the best of our knowledge, but some may still remain. Our genomes are draft genomes with several contigs for each isolate and some virulence factors might be in the gap regions ergo missing from our analyses. In addition, as the knowledge of the virulence factors is mainly based on *S. aureus*, the absence of reported virulence factors in this study does not unambiguously exclude the possibility that they are present in the NAS isolates. Through evolution the putative virulence factor gene sequences in NAS may have diversified enough from those of *S. aureus* to lead to misannotations in the automatic annotation.

## Conclusions

We found several species-specific differences in the presence of putative virulence factors in *S. aureus* and NAS species. In general, virulence factors were frequent in *S. aureus*, but each of the three NAS species also harbored several of them. None of the putative virulence factors were, however, clearly associated with the type of mastitis (subclinical/clinical). Thus, it is likely that the type of mastitis, at least when comparing subclinical and clinical mastitis with mild or moderate signs, is not a sequel of one or a few particular virulence factor(s), but a result of the interaction between bacteria and host and their distinctive genomic properties. Recent studies indicated that genes associated with the normal metabolic functions of a cell may play an important role in the virulence of a pathogen (Bosi et al., 2016). In further studies, the role of specific genes in manifestation of mastitis could be investigated by comparing virulence gene profiles of isolates from severe peracute mastitis with the profiles detected in our study.

##  Supplemental Information

10.7717/peerj.4560/supp-1 Table S1 Parameters related to the mastitis case from which the isolates of this study originate ^1^Clinical signs: 1 = no clinical signs (subclinical), 1.5 = mild clinical signs (slight changes in milk appearance), 2 = moderate clinical signs (local signs), 3 = severe clinical signs (local and systemic signs) ^2^CMT = California Mastitis Test, which estimates the milk somatic cell count. 1 = low cell count, 5 = very high cell count ^3^Data originates from the study of Taponen *et al.* ^3^ ^4^ND, not determined ^5^CM, clinical mastitis; SCM, subclinical mastitis ^6^Type strain = DSM 23656 ^*T*^ = CCUG 59809 ^*T*^.

10.7717/peerj.4560/supp-2 Table S2 General properties of *Staphylococcus* draft genome sequences used in this study ^1^CDS, the number of predicted protein-coding sequences ^2^Type strain = DSM 23656 ^*T*^ = CCUG 59809 ^*T*^. ^3^The raw sequence data for this strain has been deposited to the NCBI Sequence Read Archive (SRA) database under accession number SRP133811. ^4^Most of the raw sequence data for this strain has been deposited to the NCBI Sequence Read Archive (SRA) database under accession number SRP133811. Some raw sequence data was lost due to breakdown of a hard drive. The complete genome sequence is based on all the raw data.

10.7717/peerj.4560/supp-3 Table S3 Virulence factors identified in each *Staphylococcus* isolate For each isolate and each gene encoding specific virulence factor, the number of gene copies found by annotation is given first, and after slash, the number of copies encoding putative secreted virulence proteins, predicted by SignalP program, is given. For virulence factors found by blast analysis on whole genomes, identity (ID) and query cover (QC) percents together with corresponding *E*-value of the best hit are given.

10.7717/peerj.4560/supp-4 Table S4 Hemolysis on bovine blood agar after 23 h and 42 h of incubation at 37 °C 1 Non-hemolytic: no hemolysis detected; complete: clear zone around the colonies; incomplete: broad zone of incompletely lysed erythrocytes around the colonies, double: a clear inner zone together with a broad incomplete outer zone.

10.7717/peerj.4560/supp-5 Table S5 *In vitro* nuclease and coagulase activities of the 24 staphylococcal isolates used in the study ^1^Origin of the isolate: clinical mastitis (CM), subclinical mastitis (SCM). ^2^The deoxyribonuclease assay result is indicated at 48 and 72 h of incubation. ^3^The heat-stable nuclease assay result is indicated at 24 h of incubation. ^4^ −, negative result; (+) weak positive result; +, positive result ^5^The tube coagulase activity assay result is indicated at 2, 4 and 24 h of incubation. ND, not determined.

10.7717/peerj.4560/supp-6 Table S6 In vitro urease activities and the urease operon structure of the 24 staphylococcal isolates used in the study ^1^Origin of the isolate: clinical mastitis (CM), subclinical mastitis (SCM). ^2^The urease activity result is indicated at 24, 48 and 120 h of incubation. ^3^ −, negative result; (+) weak positive result; +, positive result. ^4^All the urease genes in a single *ureABCEFGD* operon. ^5^The urease genes are located in two different operons *ureABC* and* ureEFGD*, separated with a gap of varying length in each isolate.

10.7717/peerj.4560/supp-7 Table S7 Virulence factors identified in publicly available annotated genome sequences of S. agnetis, S. chromogenes and S. simulans of veterinary origin Y, present.
0, not present.
UC, presence uncertain.

## Additional Information and Declarations

### DNA Deposition

The following information was supplied regarding the deposition of DNA sequences: The GenBank accession numbers are provided in Table S2.
