# Supplementary material for: Comparative genome analysis of 24 bovine-associated Staphylococcus isolates with special focus on the putative virulence genes
Source: PeerJ. 2018 Mar 30;6:e4560. doi: 10.7717/peerj.4560 (PMC5880176; doi:10.7717/peerj.4560)
Supplement: Table S1 — 1Clinical signs: 1 = no clinical signs (subclinical), 1.5 = mild clinical signs (slight changes in milk appearance), 2 = moderate clinical signs (local signs), 3 = severe clinical signs (local and systemic signs) 2CMT = California Mastitis Test, which estimates the milk somatic cell count. 1 = low cell count, 5 = very high cell count 3Data originates from the study of Taponen et al. 3 4ND, not determined 5CM, clinical mastitis; SCM, subclinical mastitis 6Type strain = DSM 23656 T = CCUG 59809 T. [file peerj-06-4560-s001.docx]

| **Bacterial species** | **Isolate ID** | **Sign score**^1^ | **Body temperature (°C)^3^** | **CMT**^2,3^ | **Type of mastitis^5^** |
| --- | --- | --- | --- | --- | --- |
| *S. agnetis* | 59 | 1 | 38.5 | ND^4^ | SCM |
|  | 43 | 1 | 38.5 | ND | SCM |
|  | 6-4^T, 6^ | 1.5 | 38.5 | 5 | CM |
|  | 33 | 1.5 | 38.5 | 4 | CM |
| *S. chromogenes* | 46 | 1 | 38.5 | 4 | SCM |
|  | 92 | 1 | 38.5 | 4 | SCM |
|  | 101 | 1 | 38.5 | 3 | SCM |
|  | 121 | 1 | 38.5 | 2 | SCM |
|  | 117 | 2 | 40.8 | 5 | CM |
|  | 38 | 2 | 39.9 | 5 | CM |
|  | 22 | 2 | 39.1 | 5 | CM |
|  | 72 | 2 | 38.5 | 5 | CM |
| *S. simulans* | 102 | 1 | 38.5 | 3 | SCM |
|  | 97 | 1 | 38.5 | 4 | SCM |
|  | 78 | 1 | 38.5 | 3 | SCM |
|  | 113 | 1 | 38.5 | 3 | SCM |
|  | 15 | 2 | 39.5 | 5 | CM |
|  | 116 | 2 | 39.8 | 5 | CM |
|  | 52 | 2 | 39.2 | 5 | CM |
|  | 19 | 2 | 40.5 | 5 | CM |
| *S. aureus* | 110 | 1 | 38.6 | 3 | SCM |
|  | 112 | 1 | 38.5 | 5 | SCM |
|  | 75 | 2.5 | 40.0 | ND | CM |
|  | 9 | 3 | 41.0 | 3 | CM |
