# Supplementary material for: Comparative genome analysis of 24 bovine-associated Staphylococcus isolates with special focus on the putative virulence genes
Source: PeerJ. 2018 Mar 30;6:e4560. doi: 10.7717/peerj.4560 (PMC5880176; doi:10.7717/peerj.4560)
Supplement: Table S2 — 1CDS, the number of predicted protein-coding sequences 2Type strain = DSM 23656 T = CCUG 59809 T. 3The raw sequence data for this strain has been deposited to the NCBI Sequence Read Archive (SRA) database under accession number SRP133811. 4Most of the raw sequence data for this strain has been deposited to the NCBI Sequence Read Archive (SRA) database under accession number SRP133811. Some raw sequence data was lost due to breakdown of a hard drive. The complete genome sequence is based on all the raw data. [file peerj-06-4560-s002.docx]

| **Bacterial species** | **Isolate ID** | **Average coverage X** | **No. of contigs** | **Genome size (Mbps)** | **G+C content (%)** | **No. of CDS^1^** | **Ref.** | **Accession number** |
| --- | --- | --- | --- | --- | --- | --- | --- | --- |
| *S. agnetis* | 59 | 9 | 53 | 2.40 | 35.7 | 2353 | This study | MRYT00000000^3^ |
|  | 43 | 9 | 58 | 2.45 | 35.6 | 2384 | This study | MRYS00000000^3^ |
|  | 6-4^T, 2^ | 8 | 58 | 2.48 | 35.6 | 2449 | This study | MRYU00000000^3^ |
|  | 33 | 9 | 52 | 2.51 | 35.6 | 2457 | This study | MRYV00000000^3^ |
| *S. chromogenes* | 46 | 11 | 29 | 2.21 | 36.7 | 2116 | This study | MRYW00000000^3^ |
|  | 92 | 21 | 23 | 2.30 | 36.7 | 2231 | This study | MRYX00000000^3^ |
|  | 101 | 27 | 26 | 2.31 | 36.7 | 2260 | This study | MRYY00000000^3^ |
|  | 121 | 32 | 20 | 2.27 | 36.6 | 2196 | This study | MRYZ00000000^3^ |
|  | 117 | 30 | 16 | 2.27 | 36.7 | 2192 | This study | MRZA00000000^4^ |
|  | 38 | 20 | 34 | 2.34 | 36.8 | 2231 | This study | MRZB00000000^4^ |
|  | 22 | 26 | 19 | 2.30 | 36.7 | 2221 | This study | MRZC00000000^3^ |
|  | 72 | 17 | 24 | 2.32 | 36.6 | 2248 | This study | MRZD00000000^3^ |
| *S. simulans* | 102 | 28 | 30 | 2.63 | 35.9 | 2453 | This study | MRZE00000000^4^ |
|  | 97 | 17 | 34 | 2.70 | 35.8 | 2561 | This study | MRZF00000000^3^ |
|  | 78 | 24 | 25 | 2.55 | 36.0 | 2368 | This study | MRZG00000000^4^ |
|  | 113 | 19 | 32 | 2.59 | 36.0 | 2419 | This study | MRZH00000000^3^ |
|  | 15 | 17 | 26 | 2.70 | 35.9 | 2552 | This study | MRZI00000000^4^ |
|  | 116 | 19 | 28 | 2.60 | 37.3 | 2404 | This study | MRZJ00000000^3^ |
|  | 52 | 21 | 31 | 2.63 | 35.9 | 2449 | This study | MRZK00000000^3^ |
|  | 19 | 22 | 22 | 2.62 | 35.9 | 2451 | This study | MRZL00000000^4^ |
| *S. aureus* | 110 | 28 | 25 | 2.66 | 32.6 | 2547 | (31) | JZIP00000000 |
|  | 112 | 20 | 29 | 2.65 | 32.6 | 2469 | (31) | JZIQ00000000 |
|  | 75 | 23 | 26 | 2.65 | 32.6 | 2544 | (31) | JZIO00000000 |
|  | 9 | 18 | 31 | 2.70 | 32.6 | 2478 | (31) | JZIN00000000 |
