# Supplementary material for: Comparative genome analysis of 24 bovine-associated Staphylococcus isolates with special focus on the putative virulence genes
Source: PeerJ. 2018 Mar 30;6:e4560. doi: 10.7717/peerj.4560 (PMC5880176; doi:10.7717/peerj.4560)
Supplement: Table S5 — 1Origin of the isolate: clinical mastitis (CM), subclinical mastitis (SCM). 2The deoxyribonuclease assay result is indicated at 48 and 72 h of incubation. 3The heat-stable nuclease assay result is indicated at 24 h of incubation. 4 −, negative result; (+) weak positive result; +, positive result 5The tube coagulase activity assay result is indicated at 2, 4 and 24 h of incubation. ND, not determined. [file peerj-06-4560-s005.docx]

| **Species** | **Isolate ID^1^** | **Deoxyribonuclease (DNAase) activity^2,4^**  **48/72 h** | **Heat-stable nuclease activity^3,4^**  **24 h** | **Coagulase activity^5^** |
| --- | --- | --- | --- | --- |
| *S. agnetis* | 59 (SCM) | +/+ | + | -/-/- |
|  | 43 (SCM) | +/+ | + | -/-/+ |
|  | 6-4^T^ (CM) | +/+ | + | -/-/- |
|  | 33 (CM) | +/+ | + | -/-/- |
| *S. chromogenes* | 46 (SCM) | +/+ | + | ND |
|  | 92 (SCM) | +/+ | + | ND |
|  | 101 (SCM) | +/+ | + | ND |
|  | 121 (SCM) | +/+ | + | ND |
|  | 117 (CM) | +/+ | + | ND |
|  | 38 (CM) | +/+ | + | ND |
|  | 22 (CM) | +/+ | + | ND |
|  | 72 (CM) | +/+ | + | ND |
| *S. simulans* | 102 (SCM) | -/- | - | ND |
|  | 97 (SCM) | -/- | - | ND |
|  | 78 (SCM) | -/- | - | ND |
|  | 113 (SCM) | -/- | - | ND |
|  | 15 (CM) | -/- | - | ND |
|  | 116 (CM) | -/- | - | ND |
|  | 52 (CM) | -/- | - | ND |
|  | 19 (CM) | -/- | - | ND |
| *S. aureus* | 110 (SCM) | +/+ | + | +/+/+ |
|  | 112 (SCM) | +/+ | + | +/+/+ |
|  | 75 (CM) | +/+ | + | +/+/+ |
|  | 9 (CM) | +/+ | + | +/+/+ |
