# Supplementary material for: Comparative genome analysis of 24 bovine-associated Staphylococcus isolates with special focus on the putative virulence genes
Source: PeerJ. 2018 Mar 30;6:e4560. doi: 10.7717/peerj.4560 (PMC5880176; doi:10.7717/peerj.4560)
Supplement: Table S6 — 1Origin of the isolate: clinical mastitis (CM), subclinical mastitis (SCM). 2The urease activity result is indicated at 24, 48 and 120 h of incubation. 3 −, negative result; (+) weak positive result; +, positive result. 4All the urease genes in a single ureABCEFGD operon. 5The urease genes are located in two different operons ureABC and ureEFGD, separated with a gap of varying length in each isolate. [file peerj-06-4560-s006.docx]

| **Species** | **Isolate^1^** | **Urease activity^2,3^**  **24 /48/ 120 h** | **Urease operon structure**  ***ureABCEFGD^4^* *ureABC***  **and**  ***ureEFGD^5^*** | |
| --- | --- | --- | --- | --- |
| *S. agnetis* | 59 (SCM) | -/-/- |  | X |
|  | 43 (SCM) | -/-/- |  | X |
|  | 6 (CM) | -/-/(+) |  | X |
|  | 33 (CM) | -/-/- |  | X |
| *S. chromogenes* | 46 (SCM) | -/-/+ |  | X |
|  | 92 (SCM) | -/-/(+) |  | X |
|  | 101 (SCM) | -/-/(+) |  | X |
|  | 121 (SCM) | -/(+)/+ |  | X |
|  | 117 (CM) | -/-/(+) |  | X |
|  | 38 (CM) | -/-/(+) |  | X |
|  | 22 (CM) | +/+/+ |  | X |
|  | 72 (CM) | -/-/(+) |  | X |
| *S. simulans* | 102 (SCM) | +/+/+ | X |  |
|  | 97 (SCM) | +/+/+ | X |  |
|  | 78 (SCM) | +/+/+ | X |  |
|  | 113 (SCM) | +/+/+ | X |  |
|  | 15 (CM) | +/+/+ | X |  |
|  | 116 (CM) | +/+/+ | X |  |
|  | 52 (CM) | +/+/+ | X |  |
|  | 19 (CM) | +/+/+ | X |  |
| *S. aureus* | 110 (SCM) | (+)/(+)/+ | X |  |
|  | 112 (SCM) | +/+/+ | X |  |
|  | 75 (CM) | +/+/+ | X |  |
|  | 9 (CM) | -/(+)/+ | X |  |
